# LRP1 shedding in human brain: roles of ADAM10 and ADAM17

**DOI:** 10.1186/1750-1326-4-17

**Published:** 2009-04-16

**Authors:** Qiang Liu, Juan Zhang, Hien Tran, Marcel M Verbeek, Karina Reiss, Steven Estus, Guojun Bu

**Affiliations:** 1 Department of Pediatrics, Washington University School of Medicine, St Louis, MO 63110, USA; 2 Department of Cell Biology and Physiology, Washington University School of Medicine, St Louis, MO 63110, USA; 3 Department of Neurology, Radboud University Nijmegen Medical Centre, Donders Centre for Neuroscience, the Netherlands; 4 Department of Dermatology, Christian-Albrecht University Kiel, Kiel, Germany; 5 Department of Physiology and Sanders-Brown Center on Aging, University of Kentucky, USA

## Abstract

**Background:**

The low-density lipoprotein receptor-related protein 1 (LRP1) plays critical roles in lipid metabolism, cell survival, and the clearance of amyloid-β (Aβ) peptide. Functional soluble LRP1 (sLRP1) has been detected in circulating human placenta; however, whether sLRP1 is also present in the central nervous system is unclear.

**Results:**

Here we show that abundant sLRP1 capable of binding its ligands is present in human brain tissue and cerebral spinal fluid (CSF). Interestingly, the levels of sLRP1 in CSF are significantly increased in older individuals, suggesting that either LRP1 shedding is increased or sLRP1 clearance is decreased during aging. To examine potential effects of pathological ligands on LRP1 shedding, we treated MEF cells with Aβ peptide and found that LRP1 shedding was increased. ADAM10 and ADAM17 are key members of the ADAM family that process membrane-associated proteins including amyloid precursor protein and Notch. We found that LRP1 shedding was significantly decreased in MEF cells lacking ADAM10 and/or ADAM17. Furthermore, forced expression of ADAM10 increased LRP1 shedding, which was inhibited by ADAM-specific inhibitor TIMP-3.

**Conclusion:**

Our results demonstrate that LRP1 is shed by ADAM10 and ADAM17 and functional sLRP1 is abundantly present in human brain and CSF. Dysregulated LRP1 shedding during aging could alter its function and may contribute to the pathogenesis of AD.

## Background

Alzheimer's disease (AD) is the most common cause of dementia in elderly. The central hypothesis of AD is the amyloid hypothesis which proposes that accumulation of amyloid-β (Aβ) peptide, a small peptide with a high propensity to form aggregates in the brain, is the primary factor driving AD pathogenesis [1-3]. The low-density lipoprotein receptor-related protein 1 (LRP1) is a multifunctional lipoprotein receptor which recognizes over 30 ligands [4,5]. LRP1 was shown to bind to apolipoprotein E (apoE) [6] and Aβ, both of which play important roles in the pathogenesis of AD. It has been suggested that decreased clearance of Aβ from brain and CSF is the main cause of Aβ accumulation in sporadic AD [7]. LRP1 plays critical roles in Aβ clearance and neuronal survival [8,9]. Aβ has been shown to initially form a complex with LRP1 ligands, apoE and α2-macroglobulin (α2M), and then the complex binds to LRP1. LRP1-ligand complexes are then internalized to late endosomes after which they can either be delivered to lysosomes for subsequent degradation or be targeted for transcytosis across the BBB into the plasma [8]. Furthermore, Zlokovic and colleagues have provided evidence showing that Aβ can be transported across the BBB and be cleared from the brain after directly binding to LRP1 [8]. In neurons, a chimeric receptor that contains the intracytoplasmic tail of LRP1 promotes cell survival by preventing translocation of activated JNK to the nucleus [9]. Interestingly, it has been reported that the brains of AD patients had significantly lower LRP1 levels than that of age-matched controls and that LRP1 levels are decreased substantially with age in the brains of normal individuals [10].

LRP1 is synthesized as a precursor that migrates on SDS-gel with an apparent molecular size of 600 kD [11]. En route to the cell surface, LRP1 is cleaved into two subunits of 515 kD and 85 kD. LRP1-515 remains anchored to the membrane through noncovalent association with the transmembrane subunit, LRP1-85 [11]. The LRP1-515 contains multiple, Ca2+-dependent ligand binding domains. It has been reported that the presence of a soluble form of LRP1 (sLRP1) circulates in human plasma with electrophoretic mobility identical to that of the LRP1-515, which was recognized by anti-LRP1-515 antibodies and bound the LRP1 ligands a2M and the receptor-associated protein (RAP) [12,13]. The physiological significance of LRP1 ectodomain shedding remains undefined and the presence of sLRP1 in the central nervous system is unclear. Here we show that abundant sLRP1, capable of binding its ligands, is present in human brain tissue and cerebral spinal fluid (CSF). Interestingly, the levels of sLRP1 in CSF are significantly increased in older individuals. We also found that Aβ42 increases LRP1 shedding. Furthermore, LRP1 shedding was significantly decreased in MEF cells lacking ADAM10 and/or ADAM17. Together our results demonstrate that LRP1 shedding is regulated by ADAM10 and ADAM17 and functional sLRP1 is abundantly present in human brain tissues and CSF. Dysregulated LRP1 shedding during aging could alter its function and may contribute to the pathogenesis of AD.

## Results

### sLRP1 is present in human CSF and brain tissue, and the levels of sLRP1 in CSF are significantly increased in older individuals

**Figure 1:**
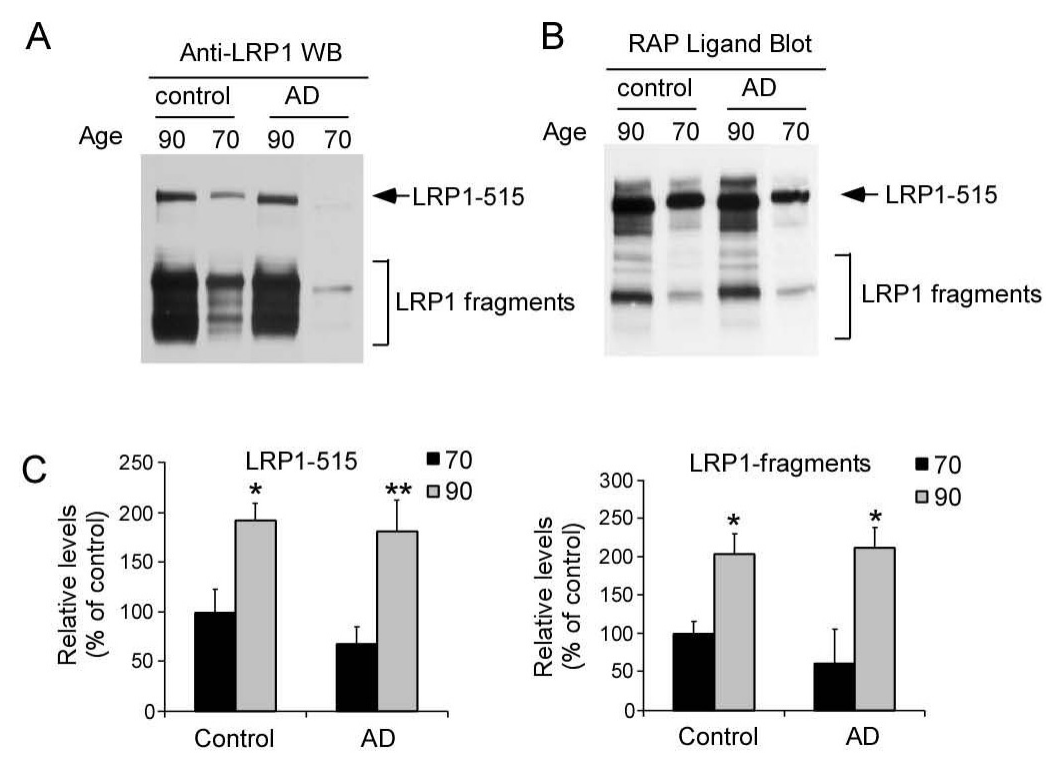
**sLRP1 is present in human cerebral spinal fluid (CSF) and the levels of sLRP1 in CSF are significantly increased in older individuals**. sLRP1 levels were measured in CSF of normal controls and AD patients by Western blot (A) and RAP ligand blot (B). (C) Band intensities of Western blot (A) were analyzed by densitometry (n = 8).

sLRP1 levels were measured in the CSF of normal controls and AD patients by Western blot (Figure 1A) and RAP ligand blot (Figure 1B). As shown in Figure 1A and 1B, the high molecular weight protein appears to be structurally and functionally related to human LRP1-515, as it is recognized by an anti-LRP antibody and binds to RAP in the RAP ligand blot. Furthermore, abundant LRP1 fragments are detected by LRP1 antibody (Figure 1A), some of which are capable of binding RAP (Figure 1B). Interestingly, both intact LRP1-515 and LRP1 fragments were significantly increased in older individuals (90 years old group) of both control and AD groups as compared to younger individuals (70 years old group) (Figure 1C), suggesting that either LRP1 shedding is increased or sLRP1 clearance is decreased during aging. However, there is no significant difference in sLRP1 levels between age-matched controls and AD patients.

**Figure 2:**
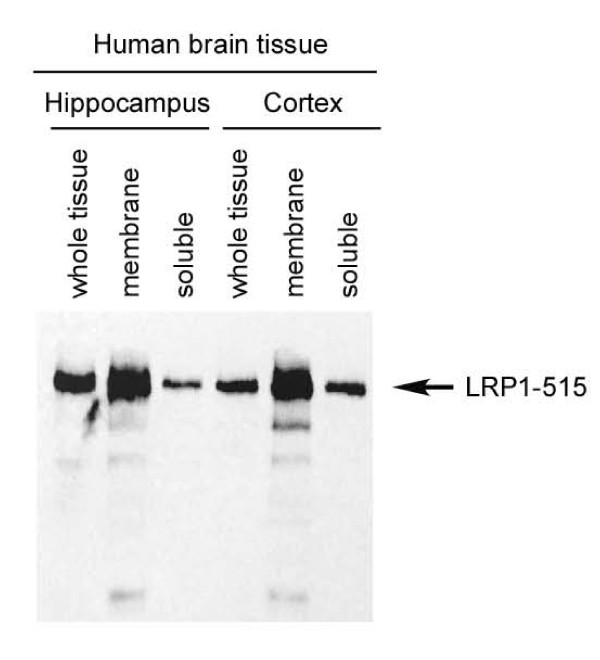
**sLRP1 is present in human brain tissues**. Cortex and hippocampus of human brain tissue were separated into membrane and soluble fractions, and were blotted wih LRP1 antibody. sLRP1 was detected in both hippocampus and cortex.

The sLRP1 levels were also analyzed in human brain tissue (from 85 years old non-AD individual) by Western blot. Cortex and hippocampus of human brain tissue were separated into membrane and soluble fractions, and were blotted with LRP1 antibody. As shown in Figure 2, sLRP1 was detected in the soluble fraction, suggesting that sLRP1 is also present in human brain tissue. The LRP1 fragments which showed in CSF samples could not be detected in human brain tissue, suggesting that intact LRP1-515 could be further cleaved by some enzymes in CSF.

### Aβ42 increases LRP1 shedding

**Figure 3:**
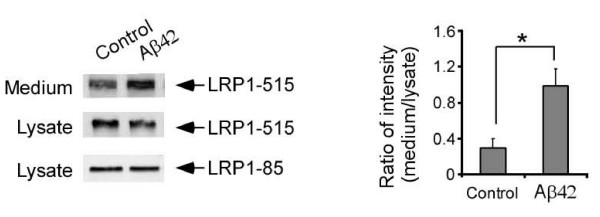
**Aβ increases LRP1 shedding**. MEF cells were treated with vehicle control or 1 μM Aβ42 for 48 h. Media and cell lysates were blotted with two LRP1 antibodies to detect either the extracellular LRP1-515 subunit or the transmembrane LRP1-85 subunit. LRP1-515 detected in the media represents sLRP1 and is increased with Aβ42 treatment. Band intensities were analyzed by densitometry from triplicate samples. The ratios of sLRP1 in the media and LRP1-85 in the lysates were compared among three treatments. Note, the relative sLRP1 levels were significantly increased after Aβ42 treatment.

To examine potential effect of pathological ligands on LRP1 shedding, mouse embryonic fibroblast (MEF) cells were treated with Aβ42 or vehicle control for 48 h. Media and cell lysates were collected and blotted with two LRP1 antibodies [9,14,15] to detect either the extracellular LRP1-515 subunit or the transmembrane LRP1-85 subunit. LRP1-515 detected in the media represents sLRP1. The ratios of sLRP1 in the media and intact LRP1 in the lysates were compared among two treatments. We found that the relative sLRP1 levels are significantly increased after Aβ42 treatment (Figure 3). LRP1 shedding is significantly decreased in MEF cells lacking ADAM10 or ADAM17.

**Figure 4:**
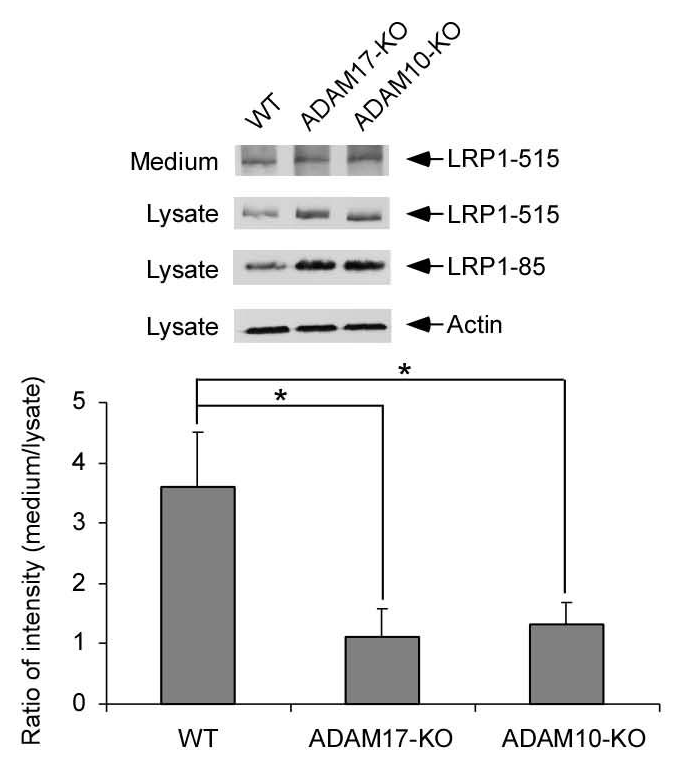
**LRP1 shedding is significantly decreased in MEF cells lacking ADAM10 or ADAM17**. LRP1 and sLRP1 levels were analyzed by Western blots as in Figure 3. Band intensities were analyzed by densitometry from triplicate samples. The ratios of sLRP1 in the media and LRP1-85 in the lysates were compared among three cell types. Note, the relative sLRP1 levels were significantly decreased in ADAM17-KO and ADAM10-KO MEF cells.

ADAM10 and ADAM17 are members of the ADAM metalloprotease family and play critical roles in ectodomain shedding of several transmembrane proteins including APP [3]. To understand how LRP1 is shed, LRP1 and sLRP1 levels were examined in ADAM17-KO and ADAM10-KO MEF cells. After one day in culture, media and cell lysates were collected and blotted with two LRP1 antibodies to detect either LRP1-515 or LRP1-85. LRP1-515 detected in the media represents sLRP1. The ratios of sLRP1 in the media and LRP1-85 in the lysates were compared among WT and KO cell types. We found that the relative sLRP1 levels were significantly decreased in ADAM17-KO and ADAM10-KO MEF cells (Figure 4). We also found that the cell associated LRP1-515 and LRP1-85 are both increased (Figure 4), indicating that LRP1 expression may be increased in the absence of ADAM10 or ADAM17.

### Over-expression of ADAM10 or ADAM17 increases LRP1 shedding, which is inhibited by ADAM-specific inhibitor TIMP-3

**Figure 5:**
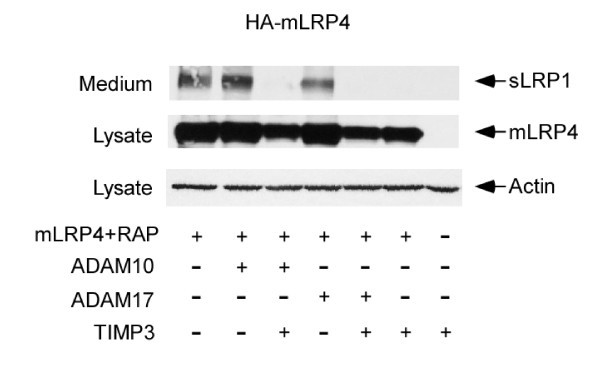
**Over-expression of ADAM10 or ADAM17 increases LRP1 shedding, which is inhibitable by ADAM-specific inhibitor TIMP-3**. CHO LRP1-null cells were transiently co-transfected with HA-tagged LRP1 minireceptor mLRP4 together with RAP, ADAM10, ADAM17, and/or TIMP-3 as indicated. The levels of sLRP1 were detected by Western blot using anti-HA antibody.

Because TIMP-3 is a natural inhibitor of matrix metalloproteinases [16], it was used to inhibit ADAM activity in this study. To examine whether forced expression of ADAM10 or ADAM17 increases LRP1 shedding, CHO LRP1-null cells were transiently co-transfected with a HA-tagged LRP1 minireceptor (mLRP4) [17] together with RAP, ADAM10, ADAM17, and/or TIMP-3. The levels of sLRP1 were then measured by Western blot using HA antibody [17]. As shown in Figure 5, over-expression of ADAM10, but not ADAM17, enhanced the shedding of mLRP4. This may suggest that endogenous ADAM17 might be sufficient for the shedding of mLRP4. Over-expression of ADAM10 or ADAM17 together with TIMP-3 completely eliminated the shedding of mLRP4. These results suggest that mLRP4 can be shed by ADAM10 and ADAM17, which are inhibited by TIMP-3.

## Discussion

LRP1 belongs to the low-density lipoprotein receptor family, the members of which share many structural and functional characteristics. The LRP1-515 subunit of LRP1 contains multiple Ca^2+^-dependent ligand binding domains and is bound to the membrane-spanning LRP1-85 subunit. Functional soluble LRP1 (sLRP1) has been detected in circulating human placenta; however, whether sLRP1 is also present in the central nervous system is unclear. Here we show that sLRP1 is abundantly present in human CSF in the forms of LRP1-515 and LRP1 fragments, which are recognized by LRP1 antibody and bind to the LRP1 ligand RAP. In human brain tissues, sLRP1 is also detected in the soluble fractions of both cortex and hippocampus. The precise nature of the LRP1 fragments needs to be further characterized by mass spectrometry using highly purified sLRP1.

Kang et al. found that the brains of AD patients had significantly lower levels of LRP1 than that of age-matched controls. Moreover, they also showed that in the brains of normal individuals, LRP1 levels are decreased substantially with age. The changes in LRP1 levels could be due to LRP1 shedding in the brain. We tested this hypothesis by comparing sLRP1 levles in normal controls and AD patients and found that the levels of sLRP1 in CSF are significantly increased in older individuals. This suggests that either LRP1 shedding is increased or sLRP1 clearance is decreased during aging. However, we did not detect significant differences in sLRP1 levels between normal controls and AD patients. This was somewhat surprising as we found that Aβ42 significantly increases LRP1 shedding *in vitro*. It is possible that increased LRP1 shedding in AD leads to its deposition in amyloid plaques [18] rather than distribution to CSF. Further studies are required to test this hypothesis.

The detection of sLRP1 in conditioned medium of MEF cells provides a model system for elucidating the mechanism of LRP1 shedding. ADAM family members are involved in ectodomain shedding of various cell surface proteins such as growth factors, receptors and their ligands, cytokines, and cell adhesion molecules [19]. Among them, ADAM10 and ADAM17, also referered to as α-secretase, are the sheddases for APP. And Hoe et al. found that TIMP3 inhibited the α-secretase (ADAM10 and ADAM17) cleavage of ApoER2, an apoE receptor [20]. In the present study using ADAM10-KO and ADAM17-KO MEF cells, we found that ADAM10 and ADAM17 are also involved in LRP1 shedding. Furthermore, forced expression of ADAM10 significantly increased the shedding of LRP1, which was inhibited by TIMP3, a natural inhibitor of ADAMs. These results demonstrate that LRP1 is likely shed by ADAM10 and ADAM17. Previous studies have already shown that LRP1 can also be cleaved by beta site of APP-cleaving enzyme (BACE1), also referered to as β-secretase, to release secreted LRP1 in the medium [21], suggesting that BACE1 cleavage may also contribute to the production of sLRP1.

The identification of the soluble forms of LRP1 in human CSF and brain tissue introduces a new dimension into the biology of this unique molecule, especially in neuropathology and aging. Further studies are required to understand the structures of the LRP1 fragments and establish their physiological role in the brain and CSF. Dysregulated LRP1 shedding during aging could alter its function and may contribute to the pathogenesis of AD.

## Conclusion

Functional sLRP1 is abundantly present in human brain and CSF, and is likely increased during aging. LRP1 shedding is mediated by ADAM10 and ADAM17 and is up-regulated by Aβ42. Increased LRP1 shedding during aging could contribute to the pathogenesis of AD.

## Methods

### Materials

Human recombinant RAP was expressed in a glutathione *S*-transferase expression vector and isolated as described previously [22]. All tissue culture media and serum were from Sigma. In-house anti-LRP1 has been described previously [9,14,15]. Peroxidase-labeled anti-mouse antibody and ECL system were from GE Healthcare. Human Aβ42 was from BACHEM. Human CSF samples are from AD patient of Braak Stage of 6 and normal controls and 8 samples from each group were analyzed.

### Cell culture

MEF cells were cultured in Dulbecco's modified Eagle's medium (DMEM) supplemented with 10% fetal calf serum in humidified air with 5% CO_2 _at 37°C. CHO LRP1-null cells were cultured in Ham's F12 medium supplemented with 10% fetal calf serum in humidified air with 5% CO_2 _at 37°C.

### Transfection

CHO LRP1-null cells were transfected with the appropriate cDNAs: LPR1 minireceptor (mLRP4), RAP, ADAM10, ADAM17 and/or TIMP3. Twenty-four hours after transfection, media was collected and concentrated, and cells were rinsed, gently scraped into PBS (pH 7.4), and pelleted. Cells were then lysed in lysis buffer, and the lysate and concentrated media were used for Western blot.

### Preparation of soluble and membrane fractions from human brain tissue

Preparation of soluble and membrane fractions has been described before [23]. Briefly, brain tissues were homogenized in ice-cold homogenization buffer (20 mM Tris(pH 8.0), 150 mM NaCl, 1 mM CaCl_2_, with EDTA-free Complete proteinase inhibitors) using a Polytron homogenizer. After two-low speed spins (1,500 × g and 10,000 × g), the homogenate was centrifuged 30 min at 100,000 × g at 4°C to separate the membrane fraction (pellet) from the soluble fraction. The pellet was resuspended in 50 mM Tris (pH8.0), 80 mM NaCl, 2 mM CaCl2, with EDTA-free Complete proteinase inhibitors. Triton X-100 was then added to both the membrane and soluble fraciton to a final concentration of 1%. The membrane fraction was passed through a 28-gauge needle and cleared by a second centrifugation at 100,000 × g to remove insoluble material.

### Western blotting and RAP ligand blotting

Cell and tissue samples were lysed on ice in lysis buffer (phosphate-buffered saline containing 1% Triton X-100, 1 mM phenylmethylsulfonyl fluoride and protease inhibitor cocktail from Roche). CSF samples were directly mixed with sample buffer. Protein concentration was determined in each sample using a Protein Assay kit (Bio-Rad). Equal amount of protein was used for SDS-PAGE. Immunoreactive bands were visualized by enhanced chemiluminescence and exposed to film. In RAP ligand blotting, blots were probed with 20 nM RAP for 1 h at room temperature, and then with RAP-specific antibody. For densiometric analyses, immunoreactive bands were scanned using a Kodak Digital Science DC290 Zoom camera and quantified using Kodak Digital Science image analysis software.

### Statistical Analysis

All quantified data represent an average of at least triplicate samples. Error bars represent standard deviations. Statistical significance was determined by Student's t-test and P < 0.05 was considered significant.

## Competing interests

The authors declare that they have no competing interests.

## Authors' contributions

QL and GB designed all the experiments. QL carried out all the studies, participated in acquisition of data, performed the statistical analysis, and drafted the manuscript. Under QL's supervision, JZ and HT carried out the transfection and Western blot study and participated in data acquisition. GB supervised QL, JZ and HT in experiments and participated in preparation of the manuscript. MMV and SE provided human CSF and brain tissues. KR provided ADAM10-KO and ADAM17-KO MEF cell lysates and media. All authors read and approved the final manuscript.
